# paraSBOLv: a foundation for standard-compliant genetic design visualization tools

**DOI:** 10.1093/synbio/ysab022

**Published:** 2021-08-30

**Authors:** Charlie J Clark, James Scott-Brown, Thomas E Gorochowski

**Affiliations:** School of Biological Sciences, University of Bristol, Bristol, UK; Nuffield Department of Population Health, University of Oxford, Oxford, Oxfordshire, UK; School of Biological Sciences, University of Bristol, Bristol, UK; BrisSynBio, University of Bristol, Bristol, UK

**Keywords:** SBOL visual, genetic design visualization, synthetic biology, Python

## Abstract

Diagrams constructed from standardized glyphs are central to communicating complex design information in many engineering fields. For example, circuit diagrams are commonplace in electronics and allow for a suitable abstraction of the physical system that helps support the design process. With the development of the Synthetic Biology Open Language Visual (SBOLv), bioengineers are now positioned to better describe and share their biological designs visually. However, the development of computational tools to support the creation of these diagrams is currently hampered by an excessive burden in maintenance due to the large and expanding number of glyphs present in the standard. Here, we present a Python package called paraSBOLv that enables access to the full suite of SBOLv glyphs through the use of machine-readable parametric glyph definitions. These greatly simplify the rendering process while allowing extensive customization of the resulting diagrams. We demonstrate how the adoption of paraSBOLv can accelerate the development of highly specialized biodesign visualization tools or even form the basis for more complex software by removing the burden of maintaining glyph-specific rendering code. Looking forward, we suggest that incorporation of machine-readable parametric glyph definitions into the SBOLv standard could further simplify the development of tools to produce standard-compliant diagrams and the integration of visual standards across fields.

## 1. Introduction

The Synthetic Biology Open Language Visual (SBOLv) standard defines a set of glyphs and conventions for visually displaying the design information of engineered biological systems (1). The use of SBOLv simplifies the communication of designs and aids both collaboration and reproducibility by removing much of the ambiguity in how core biological parts and their interactions are displayed (2, 3). SBOLv fits into a larger standards ecosystem across bioengineering (4). For example, the SBOL data standard (5–7) is used to capture detailed information about both the structure and function of a design to facilitate data exchange and enable complex design-build-test-learn workflows (8), and modeling standards like the Systems Biology Markup Language (SBML) (9) are used to simulate and test the function of possible designs before they are built.

**Figure 1.**
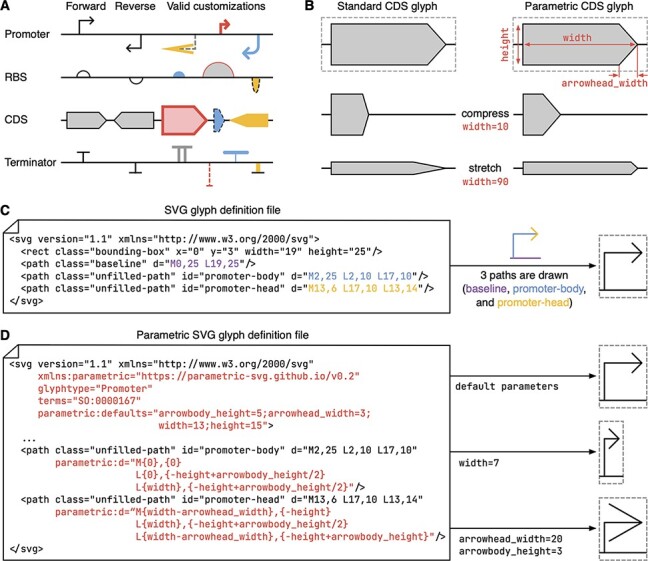
Customizable SBOLv glyphs and their representation using the parametric SVG (pSVG) format. (A) Examples of allowable aesthetic and geometric customizations of SBOLv glyphs. (B) Comparison of a standard CDS glyph and a possible parametric definition to capture the customization of glyph geometry. As changes to the width are made (e.g. compression or stretching along the x-axis), the standard glyph shape is deformed. In contrast, the parametric version can maintain key ratios (i.e. height to arrowhead_width) to ensure a similar shape is seen throughout. Parameters are shown in red and stretched versions are zoomed out. (C) Example SVG file for a promoter glyph. Styling options omitted for improved clarity. Resulting glyph shown to right and paths and their corresponding parts of the glyph shown in the color-coded version of the glyph above the arrow. The bounding box (gray dashed line) and baseline (solid horizonal line spanning the bounding box) are included to enable tools to understand how the glyph should be composed and the space it occupies. In the ‘d’ (data) attributes of the path elements in the SVG file, the ‘M’ and ‘L’ commands correspond to specifying the start point of a line path and the next point in the line path, respectively. Each of these commands are followed by two space-separated numbers corresponding to a 2D position on the canvas. (D) Example pSVG file for a promoter glyph. Elements of the file specifically used by paraSBOLv are shown in red; these supplement standard SVG elements to allow tools to interpret the file in a basic SVG format if necessary. To the right, it is shown how changes in parameters can customize the rendered glyph shape.

To support the creation of SBOLv-compliant diagrams, numerous computational tools have emerged. These include programming libraries like DNAplotlib (10, 11) and VisBOL (12, 13), as well as graphical end-user environments like SBOLDesigner (14) and SBOLCanvas (15). These tools all rely on a set of SBOL glyphs that evolve over time and so a key challenge is managing how glyphs are drawn and kept up to date. The complexity of this challenge is further increased by the SBOL standard’s permission of the customization of glyphs in many ways (Figure 1A). For example, the length of a particularly long coding sequence (CDS) might be represented by stretching the body of the CDS arrow, but not its head, as well as unique fill colors being used to visually distinguish between many different CDSs displayed in the same diagram. This required variability in geometry and basic aesthetics of each glyph makes it common for tools to use separate dedicated functions for the rendering of each glyph (10–12, 14). This simplifies the structure of the code but also leads to a significant maintenance burden as the library of glyphs changes over time and slows the propagation of updates from the standard to end-user tools. An alternative approach, which we advocate here, is to encode the glyph library in a machine-readable format that is flexible enough to capture all the information needed to tailor the rendering process through the introduction of glyph specific parameters. Parameters capture customizable features like color and line width, as well as allowable variations in glyph shape like width and height (Figure 1B). Using this approach, the rendering code of a tool can remain constant even when new glyphs are created or existing glyphs are updated.

There are many possible formats that could be used as a starting point to make this transition possible. For example, the Scalable Vector Graphics (SVG) format (Figure 1C) is one of the most pervasive for storing vector-based images and can be read by virtually all web browsers, word-processors and graphics design software, as well as a wide variety of programming libraries. However, most formats (including SVG) are designed for capturing a fixed static image and do not provide any capabilities to describe ways in which the elements within the image are allowed to be altered by a user. A rare exception is the parametric SVG (pSVG) format, which extends the capabilities of the SVG format to allow for parametric descriptions of the paths (i.e. lines and shapes) making up the image (Figure 1D). By defining paths parametrically, parameters can be provided when the image is rendered to affect the output in specific ways—precisely what is required for describing standardized glyphs where some, but not all, variations in shape forms are allowed. Furthermore, pSVG files are fully backward compatible with any software that can work with the SVG format (although without the ability for images to be customized), ensuring existing tools and libraries can make use of the same files.

In this work, we present paraSBOLv, a lightweight Python package that is designed to simplify the rendering of SBOLv diagrams by adopting pSVG files as an underlying data source for glyphs and their allowable customizations. We provide an overview of the library, the format of glyph pSVG files, the rendering pipeline and some examples of how paraSBOLv can use used to accelerate the development of SBOLv compatible visualization tools. We see paraSBOLv acting as a foundation for the growing ecosystem of SBOL compliant tools developed in Python that helps to reduce the maintenance burden on developers as SBOLv continues to evolve.

## 2. Results

### 2.1 Using the pSVG format for SBOL visual glyphs

As mentioned earlier, the pSVG file format is an extension of the SVG format, allowing for the inclusion of parametric information that can affect how paths within an image are drawn. In the context of SBOLv glyphs, parametric descriptions of geometric features are useful when glyphs can be resized in specific ways or to allow for the emphasis of certain features that correspond to part performance or function (Figure 1C). A typical SVG file is an XML file that contains elements defining the shapes or paths that should be drawn. These elements include data defining key points in these shapes/paths (e.g. the center of a circle or the points making up the start and end of line segments that create an arbitrary shape) plus styling information about how the paths and their fill should be handled. For a pSVG file, the same core SVG elements exist, but additional ‘parametric’ attributes can be included within them to allow for the points defining each shape/path to be defined in terms of user-defined parameters and basic arithmetic or trigonometric functions. This allows for changes in parameter values to affect the geometry of the shape produced. In addition, default parameters are provided so that a basic shape can always be produced if no user-defined parameters are provided.

The benefit of this approach is that pSVG files can incorporate additional information about how the shape of a glyph can be altered. The SBOLv standard already includes such guidelines for each glyph in their definitions, but this is currently encoded as free text that cannot be easily interpreted by a computer. By encoding this information directly within the files defining the set of available SBOLv glyphs, developers would be able to create tools that can immediately understand and use these allowable customizations.

**Figure 2.**
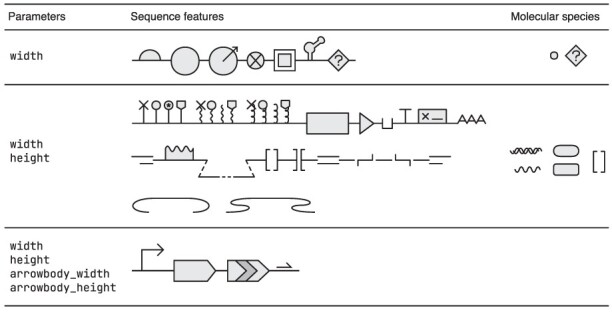
Groups of glyphs and their parameters. Sequence features and molecular species are separated, and all glyphs shown using default parameters.

With this as a goal, we developed a set of pSVG files that encompass the entire SBOLv glyph library (Data availability) (1). During this process, it became clear that glyphs often grouped into subsets with shared parameters. For example, many of the glyphs have a stem and head shape (e.g. see DNA/RNA/protein positions), while others include arrows of different forms. To ensure that parameters were consistent across the entire library, each subset of glyphs was given similar parameter names and similar terminology was adopted for parameters throughout (Figure 2). In addition, default parameters for all glyphs were chosen such that their basic (noncustomized) shapes were highly distinguishable when composed together. All pSVG glyph files are available from the paraSBOLv development repository (see Data availability).

### 2.2 The paraSBOLv Python package

Having developed a complete set of pSVG SBOLv glyphs, we next needed a way to utilize their unique capabilities. The paraSBOLv Python package was developed to provide a set of lightweight functions to load, customize and render SBOLv glyphs stored in a pSVG format. It has been designed to use matplotlib as a canvas on which to draw diagrams allowing for precise vector-based graphics that can be easily incorporated into existing visualizations and analysis scripts (e.g. by including SBOLv constructs in figures and plots) and the ability to export these diagrams to a wide range of vector and rasterized image file formats (e.g. PDF, PNG and JPEG). Internally, paraSBOLv also makes use of the svgpath2mpl package to generate matplotlib-compatible paths from an SVG definition.

**Figure 3.**
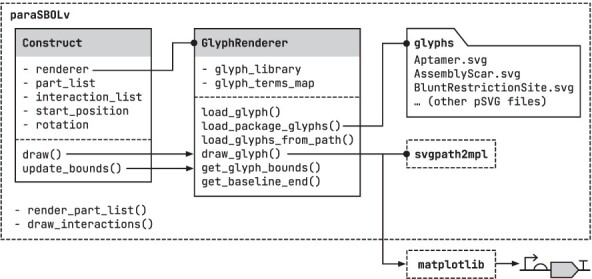
Overview of the paraSBOLv Python package. Dashed boxes represent packages. Solid boxes represent classes. The folder symbol represent directories. Arrows represent the flow of function calls between classes and packages, and lines ending in a filled circle show usage. Only key functions and class members/variables shown. Full documentation is available from the development repository (see Data availability).

Structurally, the paraSBOLv package consists of two classes and additional helper functions (Figure 3). The core rendering functionality is encapsulated in the GlyphRenderer class that can load an entire library of SBOLv pSVG glyph files and then draw them using provided customization parameters. Normally, a single GlyphRenderer object is created by an application, and this object is used multiple times to draw all the glyphs present in a diagram. To simplify this process, after drawing a glyph at a specified location (where the location is the start point of the glyph’s baseline), the GlyphRenderer object will return the bounds of the drawn glyph and the end point of the baseline, ensuring that the location of the next glyph in a design is known. While the GlyphRenderer class focuses on drawing individual glyphs, to simplify the drawing of entire genetic constructs, the Construct class is provided. This takes a GlyphRenderer object used to perform all drawing functions, a list of parts, interactions and other parameters and can render an entire construct with a single function call. Much of the rendering pipeline can be tailored based on dictionaries of attribute-value pairs provided on a per part basis that customize the way the glyph is drawn, including parameters affecting glyph shape. Specifically, when drawing each glyph, two dictionaries can be sent to alter the rendering process. The first dictionary termed ‘user_parameters’ has key-value pairs corresponding to a pSVG parameter name and its values. These parameters affect the geometry of the glyph (e.g. its width and height). The second dictionary termed ‘user_style’ has key-value pairs corresponding to each path name in the glyph with the value being a dictionary specifying the standard matplotlib styling options for that path (e.g. edgecolor and linewidth).

A core consideration throughout the development of paraSBOLv has been to keep the package small and well documented. By adopting the pSVG format for glyphs, we were able to provide a full range of rendering capabilities (including customization) in the GlyphRenderer class in less than 500 lines of code, with ∼40% of those being comments for documentation. In addition, we have also made use of continuous integration tools to simplify maintenance and ensure code quality as the package is developed (16). Specifically, we have integrated GitHub actions into the development repository to regenerate all documentation using pdoc and perform automated testing after every code commit. This approach ensures that we quickly flag introduced errors and ensure that core functionality is maintained throughout development.

### 2.3 Rapid implementation of specialized tools using paraSBOLv

When developing computational tools, two different approaches are often pursued. In one, a comprehensive, all-encompassing approach is taken where every form of functionality a user might require is ‘baked in’ by default. Common examples are tools with complex graphical user interfaces like SBOLDesigner (14), SBOLCanvas (15), Benchling and SnapGene. Such software is immediately accessible to a user but can take significant time and resources to develop to a fully functional state and are generally rigid in the way they work; it can be difficult to customize or change specific aspects to a user’s liking as workflows have a prescribed set of steps that must be followed. Alternatively, tools can be designed to only provide a small set of highly interoperable building blocks that must be pieced together by the user themself to create a specialized tool each time a new functionality is required. This approach is sometimes referred to as the ‘UNIX philosophy’ and while placing an emphasis on the user learning how to program the system, it offers the ability to rapidly create a working tool tailored to a task at hand and offers significantly greater flexibility in the final product produced.

**Figure 4.**
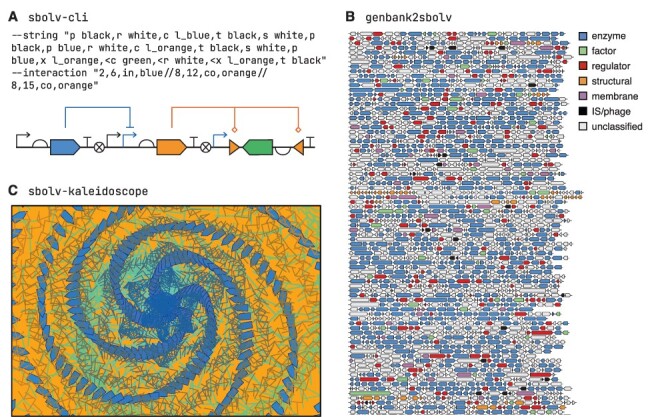
Examples of specialized visualization tools rapidly built using paraSBOLv. (A) sbolv-cli—an SBOLv Command Line Interface that converts a genetic design string into an SBOLv diagram (similar to the tool pigeon (17)). The command line arguments (--string and --interaction) used to produce the visualization are shown. Within these, ‘p’ = promoter, ‘r’ = RBS, ‘c’ = CDS, ‘t’ = terminator, ‘x’ = recombination site and a prefix of ‘<’ reverses the direction of the part. Colors are given as names with the ‘l_’ prefix denoting a lighter colored version. Interactions are given as a ‘//’ separated list where the start and end index of the part the interaction spans are first given, then the type (‘in’ = inhibition, ‘co’ = control) and finally the color. (B) genbank2sbolv—a visualization tool that generates an SBOLv visualization from a GenBank file. Long genetic designs (e.g. genomes) are split across lines to simplify viewing, and coding regions are shown in correct relative sizes with color corresponding to annotated function. Visualization shown for part of the *Escherichia coli* MG1655 genome (GenBank: U00096.3). (C) sbolv-kaleidoscope—a generative art tool where all elements of an image are customized SBOLv symbols. Code for all examples is available from the development repository (see Data availability).

ParaSBOLv falls into this second category. This does not preclude paraSBOLv acting as a foundation for building larger and more comprehensive genetic design visualization tools, but it is most powerful when used directly to rapidly create highly specialized visualization software. To demonstrate this capability, we developed several example tools that allow for a broad range of visualization tasks to be achieved with little effort (Figure 4). All tools are available at the public paraSBOLv development repository (see Data availability).

Our first tool, called sbolv-cli, demonstrates the drawing of genetic designs specified by a shorthand notation using a command line interface, similar to the tool pigeon (17) (Figure 4A). The user provides a string encoding the parts that should be present, customization information regarding each of these (e.g. color and orientation) and the interactions present between the parts using their indexes in the design (full usage details are available from the development repository). This information is parsed by sbolv-cli and a design generated that can then be rendered to file by paraSBOLv. This tool is particularly useful for users needing to generate short genetic design ‘snippets’ that can be included in larger diagrams or as part of presentations or documents.

Our second tool, called genbank2sbolv, shows how basic data processing and visualization can be combined. Taking a file in the commonly used GenBank format as input, it parses the content and produces a graphical representation (Figure 4B). This helps improve the viewers’ understanding of the overall organization of a DNA sequence in relation to the genetic parts encoded. A key feature of this tool is its ability to split the visualization across lines to improve the readability of very long and complex designs. It is also able to use specific attributes associated with each coding region to tailor the rendering process (e.g. allowing for the color to be modified in relation to the annotated function).

Our final example, called sbolv-kaleidoscope, is a dynamic generative art tool that can create unique moving artworks consisting solely of customized parametric SBOLv glyphs (Figure 4C). The user can specify parameters that tune the generative process and even provide constraints on the colors and geometric features of the glyphs that should be varied. While this tool has limited scientific value, it does demonstrate the diversity of ways paraSBOLv can be used beyond typical bioengineering use cases and exemplifies the variation that is possible in SBOLv glyphs while still being clearly recognizable as biological parts with a defined functionality.

All the tools presented above are available from the gallery of examples on the public online paraSBOLv development repository (see Data availability). Details of their specific use and available options are provided in tool-specific README files.

## 3. Conclusion

We have provided an overview of the paraSBOLv Python package and shown how its use of the pSVG format allows for a richer machine-readable description of glyphs, removing the need to write specialized rendering functions for each. This allows the core paraSBOLv package to remain lean, significantly reduces maintenance and allows for the package to make immediate use of new glyphs as they become available. Although the focus here has been on using this approach to produce SBOLv compliant visualizations, the concept of a glyph format that can capture both core geometry and allowable customizations could be easily applied to other graphical standards, such as the Systems Biology Graphical Notation (SBGN) (18), and support an opportunity for greater exchange of symbols and code between related standards.

Looking forward, we are in the process of submitting an SBOL Enhancement Proposal (1) to advocate for the use of pSVG as the core representation of each glyph within the SBOLv standard. This will ensure new glyphs capture customization information in a machine-readable format and allow tools like paraSBOLv to immediately access updates to glyphs without any change to their codebase. The use of pSVG glyphs by the SBOLv standard development process would also have further benefits; for example, allowing existing online SBOLv ontology tools to serve up pSVG files as they are needed (19) to ensure visualizations always use the most up-to-date versions of glyphs, as well as the ability to broadly automate many tasks currently performed manually by the SBOLv community (e.g. the generation of glyph tables for the website and individual sets of stencils for use in graphic design software where acceptable customization needs to also be shown). We are also in the process of transitioning our more advanced genetic design visualization tool called DNAplotlib (10, 11) to use paraSBOLv for all rendering tasks. This will remove the need to maintain low-level rendering tasks and allow for the tool to focus on higher-level functionalities (e.g. the generation of appropriate visual layouts for large and complex multimodule designs or even genomes).

Another interesting future direction for this approach is the incorporation of visualization parameters directly into SBOL data files containing the raw underlying structural and functional information about a biological design. This could be achieved by having conventions for how glyph parameters should be stored as custom annotations within the SBOL file (6). These would be then directly linked to their associated element and could be used to affect the rendering of a design. There are some additional complications with this approach due to the SBOL standard offering far greater freedom over how a biological design is structured, some of which are difficult to automatically map to a visualization; however, efforts toward this type of goal in regard to the storing of user-defined layout information has already begun with SBOLCanvas (15) leading the way.

Here, our focus has been on the development of a Python library to support the use of pSVG glyphs when rendering SBOLv diagrams. Python was chosen due to its widespread use across the sciences and growing position as a key language for computational data analysis. However, it should be noted that our use of the open pSVG format also makes it possible to use our glyph files in other programming languages. First, because each pSVG file is stored using XML, standard language-specific XML parsers can be used to extract the relevant tags (e.g. glyph paths) and their associated attributes and values. For parametric attributes the parameterized strings must first be evaluated using user-provided values or default values also stored within the pSVG file (under a ‘parametric:defaults’ attribute of the main ‘svg’ tag). Once evaluated, these strings are valid SVG paths that can then be rendered using any compatible graphics library. For example, the Cairo graphics library can render SVG strings to screen or file and is available for virtually all mainstream programming languages.

As the SBOLv standard grows to support new areas of synthetic biology and bioengineering (20–32), rethinking how we collect, manage, visualize and distribute data for supporting computational tools will become crucial (8). The paraSBOLv package and its use of the pSVG format offers a way to address some of these challenges, taking a burden off tool developers and opening new avenues for interactions between graphical standards.

## Data Availability

All the example code (stored within the ‘gallery’ directory of the development repository) and the paraSBOLv Python package are open source, released under an MIT license and publicly available at: https://github.com/BiocomputeLab/paraSBOLv.
